# Refinement in Post-Operative Care for Orthopaedic Models: Implementing a Sheep Walking Cast (SWC) for Effective Tibial Fracture Management

**DOI:** 10.3390/biomedicines12020343

**Published:** 2024-02-01

**Authors:** Ivonne Jeanette Knorr, Leonie Tix, Wenjia Liu, Steven R. Talbot, Mareike Schulz, Laura Bell, Babette Kögel, Rene Tolba, Lisa Ernst

**Affiliations:** 1 Institute for Laboratory Animal Science and Experimental Surgery, Medical Faculty, RWTH Aachen University, 52074 Aachen, Germany; iknorr@ukaachen.de (I.J.K.); ltix@ukaachen.de (L.T.); wliu@ukaachen.de (W.L.); mschulz@ukaachen.de (M.S.); bkoegel@ukaachen.de (B.K.); rtolba@ukaachen.de (R.T.); 2 Institute for Laboratory Animal Science and Central Animal Facility, Hannover Medical School, 30625 Hannover, Germany; talbot.steven@mh-hannover.de; 3 Audiovisual Media Center, Medical Faculty, RWTH Aachen University, 52074 Aachen, Germany; lbell@ukaachen.de

**Keywords:** cast, tibia defect, osteosynthesis sheep, fracture treatment, osteosynthesis plate, sheep, animal welfare, refinement, 3R

## Abstract

In the healthcare system, lower leg fractures remain relevant, incurring costs related to surgical treatment, hospitalization, and rehabilitation. The duration of treatment may vary depending on the individual case and its severity. Casting as a post-surgical fracture treatment is a common method in human and experimental veterinary medicine. Despite the high importance of sheep in preclinical testing materials for osteosynthesis, there is no standardised cast system ensuring proper stabilisation and functionality of hind limbs during the healing of tibia fractures or defects. Existing treatment approaches for tibial osteosynthesis in laboratory animal science include sling hanging, external fixators, or former Achilles tendon incision. These methods restrict animal movement for 4–6 weeks, limit species-typical behaviour, and impact social interactions. Our pilot study introduces a Standardised Walking Cast (SWC) for sheep, enabling immediate physiological movement post surgery. Seven Rhone sheep (female, 63.5 kg ± 6.45 kg) each with a single tibia defect (6 mm mechanical drilled defect) underwent SWC application for 4 weeks after plate osteosynthesis. The animals bore weight on their operated leg from day one, exhibiting slight lameness (grade 1–2 out of 5). Individual step lengths showed good uniformity (average deviation: 0.89 cm). Group housing successfully started on day three after surgery. Weekly X-rays and cast changes ensured proper placement, depicting the healing process. This study demonstrates the feasibility of using an SWC for up to 72 kg of body weight without sling hanging via ceiling mounting or external fixation techniques. Allowing species-typical movement and social behaviour can significantly improve the physiological behaviour of sheep in experiments, contributing to refinement.

## 1. Introduction

Bone fractures in the lower leg are of major socio-economic relevance in the health economy (federal health reporting: approx. 121,000 cases in Germany in 2022) [1]. In 5–10% of patients, fracture healing is impaired or absent [2]. Fracture healing can be a complex and lengthy process that places a high burden on patients [3,4,5,6,7,8]. The goals of treating tibial fractures are the restoration of mechanical stability, proper alignment of the bones, and the assurance of functional reliability of the affected limb [9,10,11]. Imperfect alignment can lead to uneven stress and wear on the joint surfaces, often associated with significant knee pain or further arthrosis. Surgical treatment can be performed through intramedullary nailing [12,13], external fixation [14], or plate fixation [15], and the specific method used depends on the type and location of the tibial fracture. Plate fixation offers excellent stability and alignment control, making it suitable for a wide range of fracture types including complex and comminuted fractures, and is therefore often used [16]. Additionally, it allows for direct visualisation of the fracture site, facilitating precise surgical management. In this context, various plate systems have been developed, including compression plates, locking plates, as well as dynamic plates, each designed for different clinical scenarios and fracture patterns. Developing new plate systems and materials is challenging due to the stringent mechanical property requirements. Researching these involves preclinical evaluation, often utilizing in vivo assessments as a step in the regulatory process for the development of new medical products. To ensure proper translation of the findings into the human situation, a suitable animal model must be used. The desirable characteristics of an animal model include evidence of similarities with humans, both in terms of physiological and pathological aspects. Since as early as the 1970s, sheep have been well studied regarding their usability for bone research (primary/secondary bone healing) [17,18,19]. In the following years, sheep were increasingly used for diaphyseal fractures, osteotomies, and osteosynthesis as a reasonable animal model [20,21].

The size and basic anatomy of the sheep skeleton are generally comparable with the human situation [22,23]. The histological appearance and remodelling activity of sheep bones closely resemble human bones [24,25]. The forces acting on the bones are comparable in general [24,26]; sheep show a similar loading pattern of body weight on the tibia and approximate bone length when fully matured. Consequently, sheep osteosynthesis models are used to mimic human bone composition and remodelling [23,24,27,28,29,30,31,32,33]. Tibial fractures or osteotomies are extensively employed as models to study fracture treatment, healing, implantable materials, and stem cell healing [30]. Immobilization of the limbs after surgical treatment is the first and safest measure in humans. In experimental veterinary medicine, the sole surgical treatment of tibial fractures is not advisable, as complete immobilization of the animals cannot be assumed for ethical reasons.

The wellbeing and species-typical behaviour of the animals should not be restricted more than necessary. The aim of post-operative treatment is to transfer as little weight as possible to the operated fracture by immobilizing the limb and minimizing shear and rotational forces. Therefore, osteosynthesis after tibial fractures in laboratory animals is to be associated with additional post-operative measures. For large experimental animals such as sheep, sling suspensions of the animal [34,35,36,37], the application of an external fixator [38,39,40], or incisions of the Achilles tendon [41] are often performed despite their adverse effects on animal welfare. Group housing is not possible if animals are suspended in a sling with limited freedom to move. The use of an external fixator not only restricts the species-typical behaviour (lying down/standing up) of the animals, but also increases the risk of infection [42] and injury. The post-operative procedures described are a substitute for the incision of the Achilles tendon, which is no longer appropriate in regard to animal welfare. Casting the hind legs is challenging due to different anatomical angles and the quadrupedal gait and cannot be used without surgical treatment such as plate implants. In this context, the hind limbs are primarily responsible for the propulsive force in the walking movement and facilitating the process of up-righting the body’s posture. To reach an effective immobilisation of the limbs, a cast must always be applied over two adjacent joints, preventing the cast from slipping [43]. Effective padding of the cast protects both the wound from pressure and the bone sites from pressure sores.

Therefore, this study aims to develop a standard cast system for adult laboratory sheep of a high body weight up to 72 kg with respect for animal welfare. This allows the animals free movement, species-typical behaviour, and timely reintegration into the group.

## 2. Materials and Methods

### 2.1. Ethical Statement

This animal study was part of an approved study by the Governmental Animal Care and Use Committee of the federal state of North Rhine-Westphalia (LANUV, North Rhine-Westphalia, Germany) (Protocol No. AZ: 81-02.04.2020.A251).

### 2.2. Animals

Seven adult (aged over 3 years) female Rhone sheep were used in this pilot study. The weight of the animals was 63.5 ± 6.45 kg. During the experiment, the sheep were housed indoors individually after surgery (separate housing) for 72 h with visual and olfactory contact with the conspecifics and later reintegrated into the group. The housing complied with the EU Directive 2010/63 [44] and the Guide for the Care and Use of Laboratory Animals [45]. The sheep were fed with a standard diet (Ssniff GmbH, Soest, Germany) and hay. They had access to water ad libitum. The housing temperature ranged between 20 and 22 degrees with a relative humidity of 55% ± 10% and a 15 times air exchange rate. The sheep had a housing condition with a light/dark rhythm of 12/12 h, starting at 7:00 with the light phase. All animals experienced an acclimatisation period of at least 14 days before surgery.

### 2.3. Study Design and Surgical Procedure

All animals underwent a single bone defect with plate fixation of the right tibia followed by casting of the hind limb. Survival of animals was either one month or six months for long-term experimentation.

**Figure 1:**
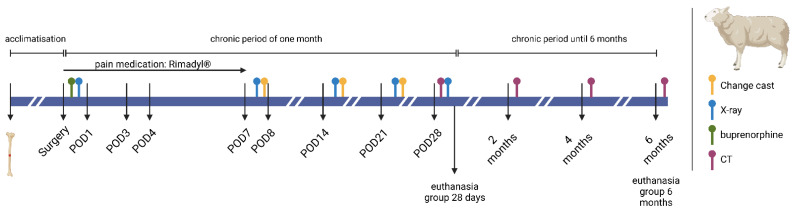
Timeline and intervention of study; This Figure was created using Biorender.com (accessed on 15 October 2023) (Agreement No. PN265EYEQV).

The precise study design is elucidated in Figure 1. After food deprivation overnight, the animals were sedated with an intramuscular injection of 0.5 mg atropine and 0.2–0.4 mg/kg xylazine. A venous catheter was placed in the left vena auricularis lateralis. After anaesthesia deepening with 2% propofol intravenously, the animals were intubated, and a gastric tube and urine catheter were placed.

The surgical field was shaved, cleaned, and disinfected with iodine. The animals were positioned on their right lateral side to obtain access to the surgical field of the right tibia. Intraoperatively, the animals were mechanically ventilated (Cato Dräger, Lübeck, Germany) using a mixture of oxygen and air and 1–2 vol% isoflurane. For analgesic treatment, 2–20 µg/kg/h of fentanyl was intravenously perfused.

After disinfection, covering the surgical field, and confirmation of surgical tolerance, a metallic drilling template, simulating the implant size, was placed on the skin for positioning. A minimum distance of 2.5 cm from both epiphyseal sides was maintained for the incision. An X-ray was taken to confirm the correct position. Subsequently, an approximately 11 cm long skin incision was made along the medial diaphyseal shaft of the right tibia. The tibia was exposed medially in the middle of the diaphysis. The tibia was dissected bluntly while preserving the adjacent nerve, and the periosteum was incised and bluntly removed using a periosteal elevator. The custom-made drilling template was used and fixed at the proximal and distal ends of the tibial shaft with two thin Kirschner wires. This template corresponded to the size of the implant. A 6 mm diameter monocortical hole was drilled in the middle to simulate the tibial bone defect. Six drill holes were placed in the longitudinal direction of the bone for the osteosynthesis plate (three proximal and three distal of the bone defect). Self-drilling screws were utilised in the plate. To ensure a good distribution of forces proximal and distal of the bone lesion, a long osteosynthesis locking plate (90 mm to 100 mm in length) with a plate thickness of 3.5 mm was also selected.

After taking depth measurements, each screw was selected in relation to length. The screw sizes ranged from 24 to 30 mm in length. The screws were inserted alternately proximal and distal to the defect. After each screw placement, an X-ray image was taken to verify the correct positioning of the screws. Subsequently, a multilayer wound closure was performed.

For infection prophylaxis, 750 mg/kg cefuroxime was intravenously injected twice a day for three days. For analgesia management, a single dosage of 5–6 µg/kg buprenorphine was given directly after surgery, and seven days post operation, 2 mg/kg carprofen was subcutaneously injected twice a day.

**Figure 2:**
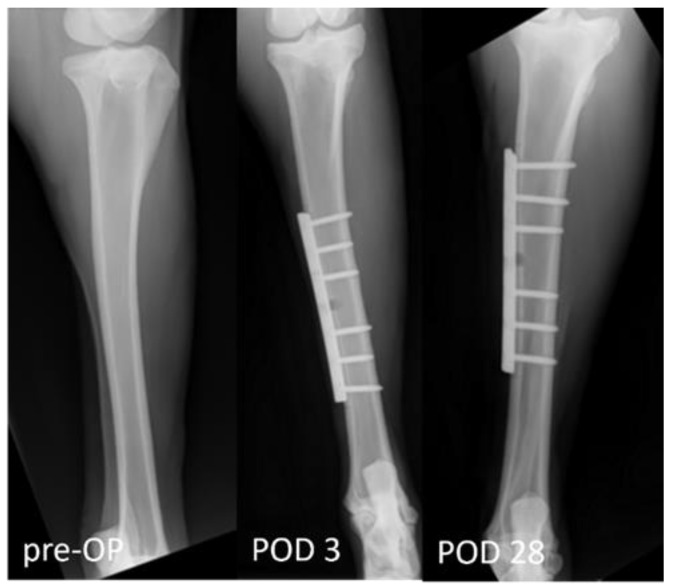
The radiographs illustrate exemplary the post-operative course. X-ray 1 shows the pre-operative tibia. X-rays 2 and 3 show the type of tibia defect and the localisation of the osteosynthesis plate at POD 3 and POD 28.

Post surgery, the health status of the operated sheep was monitored daily, based on clinical parameters. A radiographic control was performed once a week to ensure the correct plate positioning, accompanied by a cast change (Figure 2).

To check the post-operative bone healing, the fit of the implant, and the degradation quality of the test implant, computer tomography (CT) imaging of the operated limb was performed on day 28, and on months 2, 4, and 6.

The sheep were euthanised either at one or six months with 20 mL pentobarbital (160 mg/mL) and 2.98 g potassium chloride solution intravenously.

### 2.4. Cast

**Figure 3:**
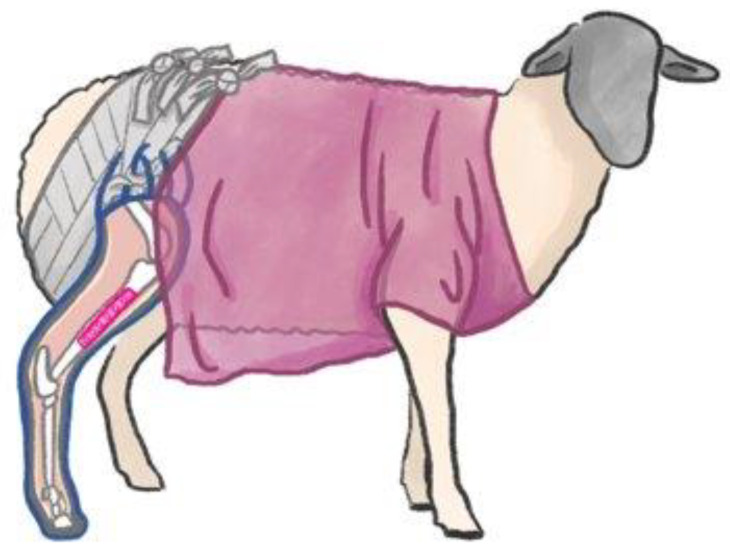
Schematic illustration of the cast structure. The animal wears a scrub. Three slings fix the cast to the back of the scrub. This illustration was created with Procreate^®^ (Version 5.3.6).

In this study, the cast was applied over the sheep’s complete limb (from the claw to the knee joint) for 4 weeks (Figure 3), securing the cast from slipping with attachment to a scrub. The initial casting was performed under general anaesthesia after surgery, and a brief anaesthesia was also administered for all following cast changes.

**Figure 4:**
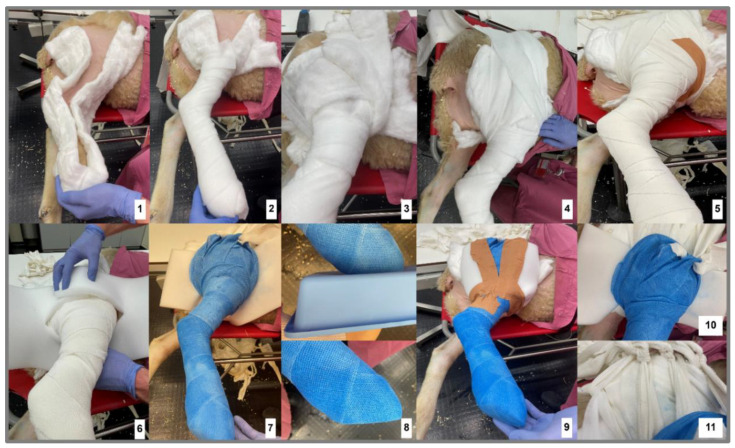
Stepwise build of the cast (**1**–**9**) and fixation (**10**,**11**).

The cast was created according to the existing veterinary literature for constructing bandages after surgical treatment [43], in detail with padding wadding (Hartmann Watte 400 g, CMC Costumer Medical Care GmbH, Sontheim an der Benz, Germany), gauze bandages (Idealbinde 10 cm × 5 m, Nobamed, Wetter, Germany), synthetic support bandages (Cellacast Xtra 10 cm × 3.6 m, Lohmann & Rauscher, Rengsdorf, Germany), tape (leucoplast tape 5 cm × 9.2 m, essity BSN medical GmbH, Hamburg, Germany), foam padding (35 cm × 18 cm), and bandage wadding (Cellona, Lohmann & Rauscher, Rengsdorf, Germany (Figure 4). First, the limb was effectively padded from all sides, including cotton padding between the claws and afterclaws to maintain blood circulation. The padding was then reinforced up to the thigh, wrapped around the back, and attached again on the hind leg from the other side (Figure 4, step 4). On top, two additional bandages were placed at a 90 degree angle to the back and attached through two fixation holes to the scrub. To prevent the cotton from slipping, a gauze bandage was also placed around the limb to fixate the dressings. The bandage was wrapped around the limb so that the knee was fully protected and padded, and the limb was flexed about 30% of the flexion capacity to shorten the leg before adding the cast material. A foam layer was placed around the thigh proximal to the knee joint to ensure that the cast material would not harm the skin in the knee area. After sufficient padding of the limbs, the cast was applied over the complete limb. Each cast bandage was wrapped in the same direction. At the upper end of the cast, three loops were created to serve as attachment points for later traction bandages.

Additionally, a loop was made on the medial side to apply traction from that direction. The cast was fitted with a plateau on the claw before it hardened to make it easier for the animals to step on. The remaining foam sticking out could then be placed around the cast on the upper leg and fixed with leucoplast tape to prevent skin irritations from the sharp cast material. The cast was attached to the scrub in the last steps (Figure 4, step 10/11). A bandage was drawn through each of the three loops. These traction bandages were consistently secured and knotted first with the 90 degree cross-bandage and subsequently with the scrub. This procedure was carried out for the three lateral and medial loops. The medial loop was tightened only to such an extent that two fists could still easily fit in between. The tensile strength of the bandages prevented the cast from slipping backwards and downward. The cast was considered adequately positioned when the knee was fully encapsulated within the cast.

### 2.5. Post-Operative Measurements

**Figure 5:**
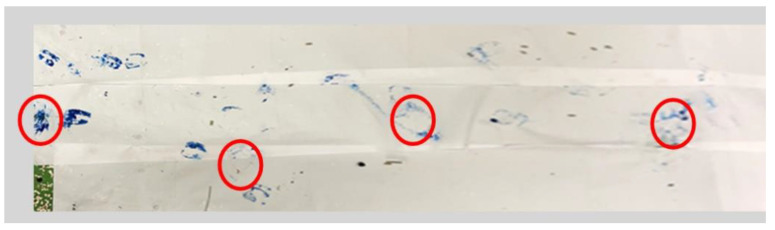
Gait analysis of footprints on wallpaper.

The post-operative function and mobility of the limb were determined via gait analysis (Figure 5). Five animals were tested once using a colour marker to evaluate the mobility of the sheep. The claws on all three healthy limbs and the tip of the cast were dipped in water-soluble finger paint. Subsequently, the animals walked over a wallpaper. The footprints were later used to analyse intensity load and step length. Lameness was scored according to an adapted protocol of Haeger et al. [34]. The degree of lameness was assessed on a scale from grade 0 (regular standing and walking) to grade 4 (no weight bearing on the limb while standing). The detailed score evaluation is presented in Table 1. Scoring was measured twice a day within the first 7 post-operative days and once a day until day 14, followed by twice a week until day 28 and once a week until the end of the experiment.

### 2.6. Statistics

Statistics and graphs were created using GraphPad Prism Version 10.0.2 (GraphPad Software, San Diego, CA, USA, www.graphpad.com (accessed on 7 November 2023). For gait analysis, a linear mixed-effect regression (lmer) was used to estimate the gait length depending on the fixed effects of step number and body weight as well as their interaction, using the lme4 and lmerTest R-packages (R version 4.3.1). The animal ID was included as a random effect to model the within-subjects correlation (repeated measures) and to adjust for individual intercepts. The importance of the animal ID was tested in a likelihood ratio test against the Null model. Results were reported as linear regression coefficients (β).

The timeline (Figure 1) was illustrated using Biorender.com (licence agreement #PN265EYEQV). Figure 2 was created with Procreate^®^ (Version 5.3.6).

## 3. Results

Table 2 shows the animals included in the study, displaying their body weight and survival. All animals reached the designated experimental end point and were humanely euthanised.

### Gait Analysis and Lameness Score

**Figure 6:**
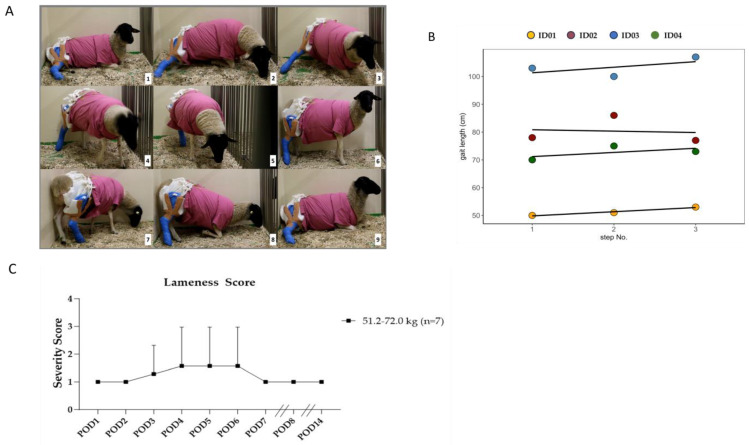
(**A**) Stepwise independent standing up/laying down from sheep with the cast. (**B**) The gait length as a function of the step number. The observed animals are represented by coloured points and individual regressions, accounting for the individual intercepts. No statistical differences in gait length were found. (**C**) Lameness scores of the sheep grouped by initial weight over a post-operative period of 28 days.

All animals showed weight bearing on the treated hind limb from post-operative day 1 (POD). Figure 6A shows the detailed free movement including the standing up and laying down of the sheep. Two videos of the sheep showing this functionality and change in body positions are provided within the Supplemental Materials for visualising the function of the cast (Video S1).

The gait length of four animals was analysed with linear mixed-effect regression, using the step number and the body weight as fixed effects. The animal ID was included as a random effect to adjust for individual intercepts. Figure 6B shows the individual gait lengths of four exemplary animals. The results of the regression are shown in Table 3. No significant difference in the gait length could be determined for the step number parameter (β_Step_ = 4.92, SE = 13.48, *p* = 0.73), the body weight (β_Bw_ = 3.03, SE = 1.22, *p* = 0.10), and the interaction of step number and body weight (β_Step:Bw_ = −0.06, SE = 0.23, *p* = 0.79). A likelihood ratio test compared the full model against the Null model, containing only the IDs as random effects. The IDs explained 92.72% of the total variance information (X^2^ = 6.9, *p* = 0.075). In conclusion, we found that the individual animals had no significant impact on the regression model. This indicates a good uniformity in gait length, with an average standard deviation of 0.89 cm.

The lameness of the animals in the first seven PODs was in general mild, being up to moderate in one case, and not exceeding a mean score of 2.3 points in any case (Figure 6C). We categorised the animals according to their initial weight class to examine the possible differences in lameness due to higher loads on the tibia defect. A significant difference (*p* < 0.01) could be observed between POD 4 and POD 6 in the lightest weight group. After this, all animals showed a mild lameness of 1 score point.

## 4. Discussion

The sheep is one of the leading animal models for tibia osteosynthesis, especially for answering biomechanical questions. The Federal Institute for Risk Assessment in Germany reports that 2671 sheep were used in biomedical research in 2021, of which 56 were used in musculoskeletal research [46].

Consequently, following the EU Directive 2010/63, improvement of animal welfare and animal models in the sense of refinements are mandated.

In our study, we were able to demonstrate that improving post-operative care results in model refinement, with a focus on species-specific needs. To obtain these goals, a fundamental knowledge of the animal model is obligate.

The ethological profile on sheep species-typical behaviour should be of interest to refine and reduce any burden. Sheep are social animals, forming stable groups known as flocks [47]. Therefore, they should never be housed without at least visual and olfactory contact with other sheep [48]. The animals in this study were housed individually for the first three post-operative days next to their conspecifics, separated by enclosure fences. We were able to reintegrate all animals 72 h after osteosynthesis to their group. Our cast system enabled the sheep to engage in their natural behaviour within the group and ensure social contact. However, even in group housing, these animals should be restricted to further speed running or jumping, making it necessary to house them in a stable and not on the field during the healing phase. The animals should only be reintegrated into known groups. Otherwise, there is a risk of rank battles between the animals when animals are reintroduced into other groups. This can lead to injuries and thus impair animal welfare. Due to their casts, it is possible that the animals may not be able to defend their rank adequately.

Sheep use ruminant digestion to ingest. For the rumination and re-chewing, the animals usually lay down for this physiological behaviour. Restrained movements such as those caused by sling-hanging [36,37] or external fixators [49] may impair their welfare: The movements of standing up and lying down are impossible with either option. Although these methods are used to prevent complete weight bearing on the implant–bone combination and minimise forces of rotation and shearing on the fracture, they interfere with the animals’ physiological behaviour. To reduce this burden, a balanced solution between post-operative fracture management and animal welfare needs to be found. Our developed cast enabled the animal to directly get up and down after surgery (shown in Figure 6A and Videos S1 and S2). We could show that this was also possible for fully mature animals with a high weight of up to 72 kg. Additionally, no complications for the defect were observed in any case, implying that the cast provided full stability.

In our study, we used plate implants to stabilise the bone defect. Fracture treatment by using an intramedullary nail is also possible but it could be associated with disadvantages such as intra-articular dislocation of the nail, loss of the implant, infections [50], or fat embolism [51]. This leads to shorter intervals in post-operative care and the need for additional medical treatment, or pain associated with increased severity for the animals. The model of severing the Achilles tendon avoids the usage of the hind limb due to induction of a painful stimulus, increasing the severity for the animals. Therefore, we believe this method is incompatible with current animal welfare aspects as more efficient alternatives exist.

The advantage of cast systems is at hand as it reduces the need for extra surgical interventions and is a non-invasive treatment after osteosynthesis. Despite other research groups reporting the usage of casts in addition to plate osteosynthesis in sheep [52,53,54], there is no standardisation for its application. In this project, we demonstrate a feasible method of cast-building after tibia osteosynthesis for adult sheep up to 72 kg in body weight. Although our study was limited to single bone defects (drilled holes), plate implants are also used for complete fracture with gaps up to 50 mm in length. In further studies, investigating the feasibility of our cast regarding more severe cases of osteosynthesis would be necessary.

Cast applications, not including the knee, may lead to stabilisation against rotation but not against sheer forces or as minimisation of weight bearing on the fracture site [55]. Short cast systems also face the problem of slipping downwards and backwards. This not only results in a lack of stability but also carries the risk, due to their rigidity when slipping, of additionally introducing shear and rotational forces into motion. To prevent slipping and reach complete stabilisation, the cast system introduced here uses four slings for adherence to the scrub. The three slings on the lateral side hold the cast high and attach it forward (Figure 4, steps 1–11). The medial sling prevents the leg from tackling the outside. During the observation, the animals showed only mild to moderate lameness during the first week after surgery and only mild lameness during the rest of the healing phase (Figure 6B). The lightest animals showed significantly higher scores in the POD 4 and 6 periods, with no significant differences after discontinuing the pain medication on POD 7 or after that. However, in the gait analysis, neither the variance in the IDs nor the body weight (BW) was significant. Due to the limited number of animals, more animals might lead to significant results, and show a correlation between step length and body weight. A mild lameness score of 1 was observed during the complete study time, indicating that lameness grade 1 belongs more to the stiffness of the cast system and not necessarily to pain-related behaviour. The gait length analysis through colour imaging (Figure 5) illustrates the step sequence and loading of the operated limb, providing an easy measure for determining and visualising individual step lengths. In Figure 6C, the animals display good uniformity within their steps, with a low standard deviation. The IDs explained 92.72% of the total variance information (X^2^ = 6.9, *p* = 0.075). In conclusion, we found that the individual animals had no significant impact on the regression model (Table 3).

## 5. Conclusions

This study demonstrates the feasibility of the SWC application as a post-surgical treatment after a tibial osteosynthesis model in sheep within a clinical context without active restriction of movement. Despite the characteristics demonstrated here, including standardisation and the positive effects on the expression of species-typical behaviour, this study has its limitations. This study is burdened by restricted sample size, impeding a definitive statistical inference concerning the weight class and the nuanced effects of animal and step size. The utilisation of casted limbs was demonstrated in this context; however, no pressure measurements were conducted to ascertain the degree of load during weight bearing. It shows evidence that species-typical behaviour is significantly less restricted through this cast than through other methods such as sling suspensions or other restraining techniques. Nevertheless, stress assessments, including cortisol and corticosterone analyses, should be incorporated in future studies to quantify these effects more explicitly.

## Figures and Tables

**Table 1:** Lameness score grading from 0 to 4 score points.

Lameness	Score Points
Slight relief of the leg during movement	0
Slight relief of the leg during movement and in a standing position	1
Obvious lameness and frequent relieving in the standing position	2
Only minor load in motion and standing position	3
No load was observed in motion or standing	4

**Table 2:** Overview of the included animals with their group assignment, body weight, and days of survival.

Animal	Body Weight (kg)	Survival (Days)	Animal	Body Weight (kg)	Survival (Days)
**#ID01**	51.2	28	**#ID04**	59.4	184
**#ID02**	64.0	28	**#ID05**	63.0	183
			**#ID06**	72.0	28
**#ID03**	64.0	28	**#ID07**	70.6	28

**Table 3:** Coefficients from the linear mixed-effect regression analysis of the gait length.

	Estimate	SE	Df	*t*-Value	*p*-Value
(Intercept)	−102.83	71.63	2.69	−1.44	0.26
Step	4.92	13.45	6.00	0.37	0.73
Bw	3.03	1.21	2.69	2.49	0.10
Step/Bw	−0.06	0.23	6.00	−0.28	0.79

## Data Availability

Data are contained within the article and supplementary materials.

## Supplementary Materials

The following supporting information can be downloaded at: https://www.mdpi.com/article/10.3390/biomedicines12020343/s1.
